# Exploitation of Gene Expression and Cancer Biomarkers in Paving the Path to Era of Personalized Medicine

**DOI:** 10.1016/j.gpb.2016.11.005

**Published:** 2017-08-13

**Authors:** Hala Fawzy Mohamed Kamel, Hiba Saeed A. Bagader Al-Amodi

**Affiliations:** 1 Biochemistry Department, Faculty of Medicine, Umm AL-Qura University, Makhha 21955, Saudi Arabia; 2 Medical Biochemistry Department, Faculty of Medicine, Ain Shams University, Cairo 11566, Egypt

**Keywords:** Personalized medicine, Gene expression, Targeted therapy, Predictive biomarkers, Prognostic biomarkers

## Abstract

Cancer therapy agents have been used extensively as cytotoxic drugs against tissue or organ of a specific type of cancer. With the better understanding of molecular mechanisms underlying carcinogenesis and cellular events during cancer progression and metastasis, it is now possible to use **targeted therapy** for these molecular events. Targeted therapy is able to identify cancer patients with dissimilar genetic defects at cellular level for the same cancer type and consequently requires individualized approach for treatment. Cancer therapy begins to shift steadily from the traditional approach of “one regimen for all patients” to a more individualized approach, through which each patient will be treated specifically according to their specific genetic defects. **Personalized medicine** accordingly requires identification of indicators or markers that guide in the decision making of such therapy to the chosen patients for more effective therapy. Cancer biomarkers are frequently used in clinical practice for diagnosis and prognosis, as well as identification of responsive patients and prediction of treatment response of cancer patient. The rapid breakthrough and development of microarray and sequencing technologies is probably the main tool for paving the way toward “individualized biomarker-driven cancer therapy” or “personalized medicine”. In this review, we aim to provide an updated knowledge and overview of the current landscape of cancer biomarkers and their role in personalized medicine, emphasizing the impact of genomics on the implementation of new potential targeted therapies and development of novel cancer biomarkers in improving the outcome of cancer therapy.

## Introduction

Advanced progression in genomics has highlighted particular molecular events that lead to cancer such as DNA mutations, gene amplifications, and chromosomal rearrangements [1]. Novel cancer drugs may target these specific molecular events or even particular cell signaling molecules, yielding unprecedentedly unique anti-cancer activity [2]. Gene expression signatures have been implemented to tailor adjuvant therapy among common cancers [2], [3]. As a result, a personalized approach has replaced the “one size fits all” approach [3]. The advent of tremendous parallel sequencing is responsible for a fundamental change in discovery of biomarkers and design of clinical trials on the path to “precision medicine” or to the simply called “biomarker-driven anticancer therapy” [4]. Biomarkers are diversely defined in literature. Gallo et al. [5] outlined most of the definitions and defined biomarkers as “*any biological substance which could be measured within the body and may affect interpretation or prediction of the incidence or outcome of any disease*”. However, there is a debate involving the qualification [6], [7]. Therefore, Gallo et al. [8] has reported later a related definition involving biomarkers as “*any substance or molecule which may be measured in bio-specimen and that may be associated with health-related outcomes*”. From our point of view, this definition is too wide and should involve the specific association of biomarkers with the patient’s clinical outcome as well. Therefore, it is quite acceptable that the better definition was documented by the Biomarkers Definitions Working Group as “*it is a biological molecule that is fairly evaluated as an indicator of normal physiological, pathological processes or pharmacological responses to a therapeutic intervention*” [9]. This definition, which is more than a decade old, is considered comprehensive and sufficiently broad to include all the applications of current biomarkers [9]. In addition, biomarkers can be defined at molecular or cellular levels (DNA, RNA, and protein), utilizing biological specimens (plasma, serum, or urines), tissues, or radiological assessments [10].

**Figure 1:**
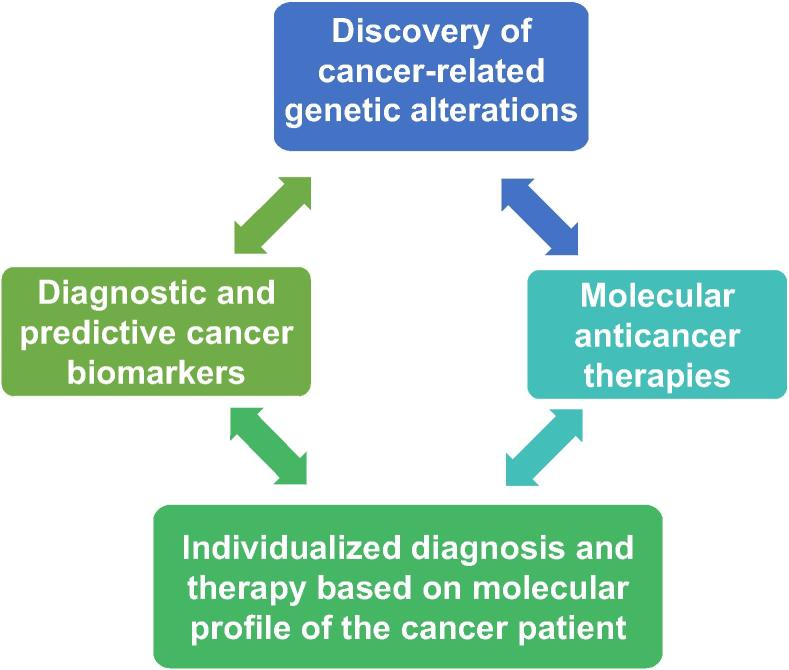
**Paradigm for exploitation of genomics in personalized medicine and therapy** The paradigm for exploitation of genomics in personalized medicine and therapy includes discovery of the genetic alterations involved in cancer initiation or progress, identification of promising predictive and prognostic biomarkers to select the appropriate anti-cancer therapy, and finally individualized diagnosis and therapy for each patient based on molecular profile for such patient.

Recent advance in personalized medicine accordingly requires development of indicators or markers that help when choosing the suitable patients to treat with such therapy, and which therapy will be mostly effective for a patient [11]. Genomic studies and extensive investigations in term of the discovery and validation of novel biomarkers, as well as development of targeted molecular anti-cancer therapy may be the key stone for development of personalized medicine. The paradigm for exploitation of genomic medicine in personalized medicine and therapy is illustrated in Figure 1.

## Terminology, discovery, and applications of biomarkers

Cancer “tumor” is considered as a disease that includes alterations of DNA at cellular level, and such alterations within tumor could be measured [12]. Biomarkers can be classified into three broad categories, *i.e.*, DNA biomarkers, DNA tumor biomarkers, and general biomarkers [13]. DNA directs synthesis of proteins that are needed for cellular structure or functions, thus genetic information coded within the DNA requires stability [14]. DNA biomarkers include variations at the sequence level of DNA, such as insertions, deletions, single nucleotide polymorphisms (SNPs), and short tandem repeats [15], [16], [17]. The most commonly utilized type of DNA alterations are SNPs, which are diallelic for most of the applications, producing three possible genotypes [18].

Distinct from DNA biomarkers, DNA tumor biomarkers represent the biomarkers specific to a particular cancerous tumor [15]. These biomarkers could be considered as powerful tools to improve the cancer treatment outcomes [19] and reduce cancer-associated deaths when they are properly used for early cancer detection and therapeutic strategy selection or, identification of subgroups of patients who might respond to the given treatment [20]. In addition, DNA tumor biomarkers could be used for prognosis or prediction of the overall outcome of a patient [21]. On the other hand, general biomarkers may be less specific to a particular cancer; hence less utilized in prognosis or prediction of outcome of therapy [13].

Prognostic biomarkers aim to predict the disease progression, whereas predictive biomarkers aim to detect the treatment response [22], [23]. Randomized, controlled data are needed to clearly identify and differentiate between predictive and prognostic biomarkers due to misleading results of single-arm studies [24], [25].

Development of new biomarkers involves multiple stages, starting initially by discovery and preclinical exploration in basic studies, then validation of the biomarker through clinical studies to identify its potential capacity for establishment of disease whether retrospectively or prospectively, and followed by clinical implementation [26]. The aim of the whole processes is to establish a clinically reliable biomarker in order to support the decision-making and to improve outcome of the patient [27]. Applying biomarkers on the basis of invalid or poorly-defined surrogate endpoints could lead to failure in achieving the required predicting power. On the other hand, few prognostic gene-expression signatures have been validated in previous studies in spite of being trained using archived specimens [28].

Pepe et al. [29] postulated a structured phased-model for discovery, evaluation, and validation of biomarkers, which is analogous to the model used in drug development. The model has been further adopted and modified by others [30], [31]. The structured phased-model includes five phases. In phase 1, preclinical studies are performed for identification and exploration of potential biomarkers, whereas validation of clinical assay is conducted in phase 2 to determine its potential capacity to establish the disease. Subsequently, retrospective and prospective studies are performed in phase 3 and phase 4, respectively. Finally, cancer control studies are performed in phase 5 through population screening [29].

**Figure 2:**
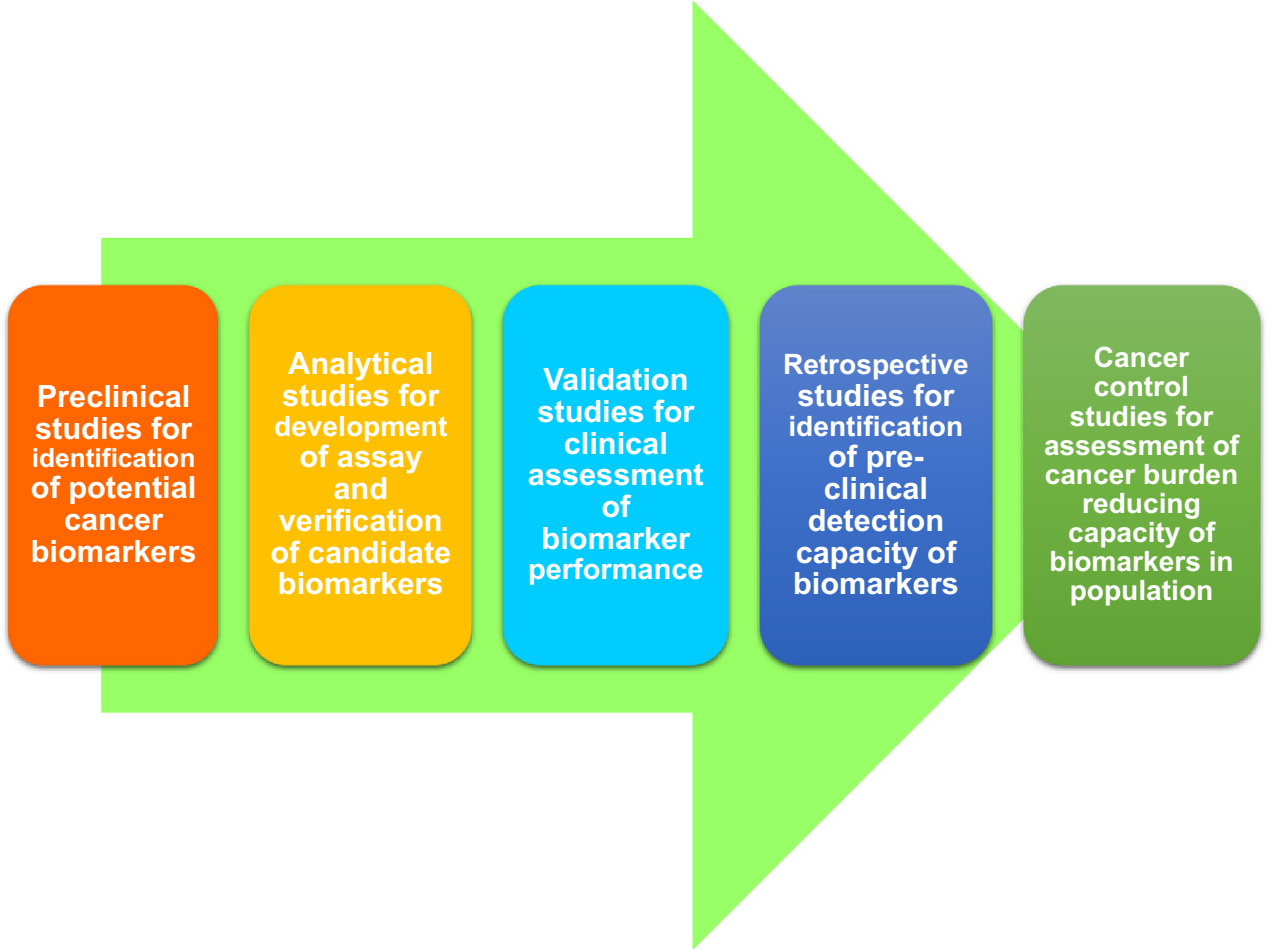
**Steps of identification and validation of potential cancer biomarkers for implementation in clinical practice** Steps involved from identification to implementation of cancer biomarkers include preclinical studies for identification of potential cancer biomarkers, analytical studies for development of assay and verification of candidate biomarkers, validation studies for clinical assessment of biomarker performance, retrospective studies for identification of pre-clinical detection capacity of biomarkers and eventually, cancer control studies for assessment of cancer burden reducing capacity of biomarkers in population.

The shortage of the process of standardized validation is the biggest challenge for adoption of biomarkers in personalized medicine practice, which may be on account of the heterogeneity of tumor kinds, therapies, and the testing process itself [32], [33]. Validation studies may also be bounded by many factors, such as small datasets, long duration required for achieving end-points, statistical errors, high costs, and the bias inherent within retrospective analysis [34]. New biomarkers tests may be commercially applied prior to completing the hard validation process; a paradigm that may exist in spite of the stark contrast of new drug development process. Therefore, rigorous demonstration of efficacy and safety are mandatory before approval of any drug for commercialization [34], [35]. The steps involved from identification of potential cancer biomarkers to implementation of cancer biomarkers in clinical practice are summarized in Figure 2.

## Techniques used for biomarker identification

The rapid development of high-throughput methods enables the integrated omics technologies, that include broad-spectrum platforms as genomics, transcriptomics, proteomics, epigenomics, and metabolomics analyses [36]. These techniques allow the detection of mutations, gene profiles, microRNA, and protein expression patterns, as well as epigenetic changes like DNA methylation and histone modifications [37]. The first draft of the human genome was completed in February 2001 using capillary sequencing technology developed by Lander et al. [38]. Subsequently, other sequencing platforms had been developed based on pyrosequencing, single molecule real-time technology, or nanopore technology (termed as third-generation sequencing) [39]. Sequencing and genomic studies enable the identification of genetic alterations that could be linked to cancer occurrence and progression [40], as well as variability in patients’ response to targeted therapies, survival, and outcomes [41]. Genomic alterations could be detected also through massively parallel sequencing [42].

Proteomics-based techniques include mass spectrometry, liquid chromatography, tandem mass spectrometry, protein arrays, and antibody assays [43]. Proteomics also plays a role in the development of personalized medicine by enabling the detection of protein biomarkers [44]. Proteomics analyses allow quantitative and qualitative assessments of protein expression changes related to cellular responses, thus providing very efficient tool for monitoring targeted therapy [45].

Moreover, transcriptomics analyses examine the level of gene expression as the proxy of gene activity [46]. Assessment of the gene expression profiles may explicate the molecular aspects of cancer progression, thus similarities and variations of gene expression profiles of cancer patients could be explored as potential biomarkers [47]. In addition, microarrays also have been implemented for evaluation of gene expression profiles, whereas RNA-sequencing can differentiate between variable isoforms, providing single base resolution for each transcript and allowing large ranges of dynamic expression [48].

Epigenetic changes, including modification of histones, chromatin, or DNA methylation status, may affect the gene expression patterns without alteration of DNA sequences [49]. Cancer cells are liable to epigenetic changes [50]. Techniques used for identification of histone modifications include DNA sequencing technologies and DNA microarrays coupled with chromatin immunoprecipitation, while DNA methylation status could be identified through bisulfite treatment and sequencing [51]. Epigenetic factors have been considered as biomarkers for some cancers [52]. For example, methylation signatures of selected genes, such as *ALX1* in non-small-cell lung cancer (NSCLC) and *RASSF1A* in prostate cancer, were found to be prognostic and correlate with relapse-free survival of the patients [53], [54]. Moreover, histone modification profiles analysis also revealed association with tumor metastasis and aggressiveness [55], [56]. Integrated “omics” analyses provides better understanding of the key crucial molecules in cancer development, and enables identification of prognostic and predictive cancer biomarkers for better assessment and follow up of cancer patients [57].

## Using biomarkers for implementation of personalized medicine

For traditional clinical procedure with cancer patients, the selection of appropriate chemotherapy is based mainly on histopathological assessment of the tumor and the primary organ from which the tumor originates [16]. Molecularly, tumorigenesis involves genetic abnormalities and aberrations in a high number [58]. Some mechanisms, such as oncogenes, existed in all neoplasia types [59]. By 2010, more than 50,000 research articles on the performance of cancer biomarkers have been reported [60]. Biomarkers utilization is critical for early diagnosis, stratification of patient, staging, prognosis, as well as evaluation of drug efficacy and toxicity and disease risk. Ideally, biomarkers should be specific and sensitive, with their concentration proportional to tumor burden to reflect the clinical stage of the disease and patient response to the treatment [61].

Biomarkers have been categorized mainly into predictive and prognostic markers, although some biomarkers might be applied for both purposes, such as methylation of *MGMT* promoter [62] and circulating tumor cells (CTCs) [63]. Genetic variability and specific polymorphisms may predispose to susceptibility to certain types of cancers, and in the response to certain treatment. Identification of these polymorphisms could rationale the use of appropriate treatment for suitable patient, fostering the entrance into the era of personalized medicine [15]. The choice of targeted therapy depending on genetic analysis is essential to treat patients carrying specific genetic aberrations for a potentially successful outcome [64].

The emergence of “omics” technologies, such as genomics, epigenomics, transcriptomics, proteomics, and metabolomics, may be the backbone toward the discovery of novel prognostic and predictive biomarkers for cancer patients [65]. Moreover, validation of thee biomarkers would consequently pave the road toward personalized medicine. Prognostic biomarkers currently available are listed in Table 1, whereas predictive biomarkers for selected cancers as well as their clinical utilities and significances are summarized in Table 2.

**Table 1:** **Prognostic biomarkers for selected cancers, their clinical utility, and significance**

**Cancer**	**Biomarker**	**Clinical utility and significance**	**Refs.**
Breast cancer	PR	PR-positive patients having higher survival rate than PR-negative patients	[171]
	ER	ER-positive patients having better survival than ER-negative patients	[172], [173]
	*BRCA1*	High *BRCA1* expression indicating worse prognosis for untreated patients	[174]
	*HER2*	Patients with HER2-positive tumors having worse prognosis and more aggressive cancer than HER2-negative patients	[175]
	MammaPrint	A 70-gene assay used to stratify patients into groups with high or low risk for relapse	[74]
	Oncotype DX	A 21-gene multiplex assay used for determining recurrence score	[176]

Colorectal cancer	CEA	Elevated serum levels of CEA associated with poor prognosis in patients	[177], [178], [179]
	LOH at 18q	Associated with metastasis and poor prognosis in patients.	[180]

Prostate cancer	*BRCA2*	Patients carrying *BRCA2* mutations having an increased cancer risk and poor prognosis	[181]
	CTCs	Increased CTCs in peripheral blood associated with poor prognosis	[182]
	*PSCA*	High *PSCA* expression correlated with higher stage, metastasis, and poor prognosis	[183]
	uPA	Elevated serum level and increased expression of uPA associated with occurrence of bone metastasis of prostate cancer	[184]

Non-small cell lung cancer	*BRCA1*	High *BRCA1*expression conferring worse prognosis in untreated patients	[185]
	*TP53*	High *TP53* expression indicating poor prognosis in untreated cases	[186]
	*KRAS*	*KRAS* mutation associated with poor prognosis, worse OS, and shorter disease-free survival	[186], [187]
	*RRM1*	High *RRM1* expression conferring better prognosis in untreated patients	[188]

*Note*: *BRCA1*, breast cancer 1 gene; *BRCA2*, breast cancer 2 gene; CEA, carcinoembryonic antigen; CTC, circulating tumor cells; EGFR, epidermal growth factor receptor; ER, estrogen receptor; GIST, gastrointestinal stromal tumor; HER2, human epidermal growth factor receptor 2; KRAS, Kirsten rat sarcoma viral oncogene; LOH, loss of heterozygosity; OS, overall survival; PR, progesterone receptor; PSCA, prostate stem cell antigen; RRM1, ribonucleotide reductase messenger 1; uPA, urokinase-type plasminogen activator.

**Table 2:** **Predictive biomarkers for selected cancers, their clinical utility, and significance**

**Cancer**	**Biomarker**	**Clinical utility and significance**	**Refs.**
Breast cancer	PR	High PR expression predicting beneficial response to tamoxifen therapy	[189]
	ER	High cellular ER expression predicting benefit from tamoxifen-based chemotherapy in node-negative patients	[114], [190]
	*BRCA1*	High *BRCA1* expression predicting response to chemotherapy	[191]
	*HER2*	Overexpression of *HER2* predicting response to treatment with trastuzumab as an adjuvant therapy or in the metastatic cases	[175], [192]
	Akt kinase isoform	Akt kinase isoforms and activity predicting response to trastuzumab-based therapy in HER2-positive metastatic cancer patients	[193]

Colorectal cancer	LOH at 18q	Predicting benefit from 5-FU based adjuvant chemotherapy	[180]
	*EGFR1*	*EGFR1* amplification predicting response to anti-EGFR1 antibody therapy	[194]
	*KRAS*	*KRAS* mutation negatively predicting benefit from EGFR-targeted therapy	[152], [195], [196]

Non-small cell lung cancer	*BRCA1*	High *BRCA1* expression predicting resistance to chemotherapy	[197]
	*TP*53	High *TP53* expression predicting sensitivity to cisplatin; *TP53* mutations predicting resistance to cisplatin	[186], [198]
	*KRAS*	*KRAS* mutation predicting lack of response to adjuvant chemotherapy in early disease and resistance to treatment with EGFR-targeted or TKI in advanced disease	[199]

*Note*: *BRCA1*, breast cancer 1 gene; EGFR1, epidermal growth factor receptor 1; ER, estrogen receptor; 5-FU, fluorouracil; HER2, human epidermal growth factor receptor 2; KRAS, Kirsten rat sarcoma viral oncogene; LOH, loss of heterozygosity; PR, progesterone receptor; TKI, tyrosine kinase inhibitor.

## Prognostic biomarkers

Prognostic biomarkers are used to monitor anti-cancer therapy, to assess the cancer grade, and to detect occurrence of remission or recurrence in individual patients [66]. Prognostic biomarkers could be linked to certain type of cancers by identifying the alteration in protein-coding genes or DNA methylation, the existence of polymorphisms, or by discovering microRNAs (miRNAs) or CTCs [67]. Mutations in genes encoding proteins that are involved in DNA repair, such as breast cancer 1 gene (*BRCA1*), breast cancer 2 gene (*BRCA2*), *TP53*, and *Ataxia-Telangiectasia mutated* (*ATM*), expose patients to an elevated risk for breast cancer [68]. Aberrations of these genes could be inherited. For instance, mutation in *BRCA2* in one allele impairs BRCA2 protein synthesis and inactivates repairing system [69]. Thus, individuals carrying alterations in the aforementioned genes are at risk for breast cancer and should undergo regular screening examinations [70]. Additionally, mutations in the genes encoding glutathione *S*-transferase (*GSTP1* and *GSTM1*) and *G158A* polymorphism in prostate-specific antigen gene may increase risk for prostate cancer [71]. Individuals carrying constitutive mutations in the gene encoding adenomatous polyposis coli (*APC*) are at risk for familial adenomatous polyposis, an autosomal dominant type of inherited genetic disorder that is characterized by increased possibility of polyps and cancer of the gut, such as colorectal cancer (CRC) [72].

A quick detection of genetic mutations in the pathogenesis of complex genetic disorders is facilitated by the genome-wide association studies and discover of various pharmacogenetic biomarkers [15]. Gene expression analysis using MammaPrint Symphony is another example for the discovery of prognostic markers in breast cancer. A 70-gene panel enables a fairly dynamic assessment of neoplastic process, allowing categorizing patients according to the high or low risk for relapse [73], [74], [75]. Together with the molecular subtypes, the information produced by these analyses allows the oncologists to select the proper chemotherapy and hormonal therapy. Nguyen et al. [76] compared the performance of microarray-based print tests and clinical assessment in cancer subtyping and evaluated the therapy response of breast cancer patients. They reported that difference in the expression levels of *BCL2*, *GRB7*, *KRT6B*, *KRT17*, and *CDH3* in breast cancer patients prior to the therapy could predict the outcomes. Given the advantages in analyzing gene expression for prediction of response to therapy, print tests show great value for personalization of cancer therapy and assessment of the possible benefits of applied therapy [76].

In addition, presence of miRNA molecules could be correlated to certain types of cancer, such as hepatocellular carcinoma (HCC) [77], and could be applied as prognostic biomarkers for multiple myeloma [78] and renal cell carcinoma [79]. Interestingly, overexpression of miR-362-3p results in the cell cycle arrest and restrains migration of cancerous cells, leading to better prognosis with increased disease-free survival for the CRC patients [80].

Alterations in DNA methylation may also be considered a good prognostic biomarker. Hypermethylation would block the promoter sites of oncogenes or tumor suppressor genes in cancer tissues, thus leading to the loss of expression or alterations of the genes [81].

In addition, the appearance of CTCs in blood may also be considered prognostic factors as well. Cells originating from the tumor may be released into circulation after passing through the vascular bed and vessel wall [82]. Chang et al. [83] reported that about 106 tumor cells per gram of cancer mass that come in contact with vascular wall and blood are released or separated from tumor mass. It was postulated that the presence of CTCs in the peripheral blood is positively and strongly associated with occurrence of metastasis and secondary tumor in other tissues [82]. Therefore, existence of few CTCs or even one cell per 10 ml of blood may indicate poor prognosis [84].

## Predictive biomarkers

Predictive biomarkers play an important role in providing data about the expected and possible response to a given therapy [85]. Moreover, they may help consolidate the decision making for the given therapy [86]. Kirsten rat sarcoma viral oncogene (*KRAS*) gene mutation is one of the alterations in the occurrence of CRC, which may occur very early in the process of carcinogenesis. The earliest report supporting the relationship between the *KRAS* mutations and the progression of colorectal carcinogenesis was published in 1988 [87]. The mutation status of *KRAS* gene in the codons 12 and 13 was documented as one of the predictive markers in the assessment of the competency of targeted therapy for patients of progressive CRC, when using cetuximab or panitumumab as therapeutic monoclonal antibodies [88]. These monoclonal antibodies target epidermal growth factor receptor (EGFR) by directly acting against the extracellular domain. EGFR cascade enhances growth and survival of CRC cells through signaling molecules including MAPK, PIK3, and JAK/STAT [85]. Implementation of the targeted therapy results in the suppressed progression of cancer cells and elevated apoptotic rate. Conversely, absence of mutations in codons 12 or 13 of the *KRAS* gene serves as a valuable positive predictive biomarker [86]. It should be noted that patients showing increased expression of EGFR without mutations in codons 12 and 13 of *KRAS* may exhibit no or worse response to the aforementioned therapy, especially if they also carry mutations in codons 61 or 146 of *KRAS*, or mutations in the *BRAF* gene [88].

*ERCC1*, a gene encoding an essential protein for DNA repair, is another predictive biomarker for response to targeted therapy in hepatic and lung cancer. It has been reported that high *ERCC1* expression is correlated with a cisplatin resistance in patients with HCC and in patients with NSCLC [89]. In addition, overexpression of *mTOR* and *c-erb-B2* may indicate tumor aggressiveness as a result of the presence of these genes in hepatic carcinogenesis [89].

Moreover, CTCs could be considered as a valuable predicative tool as well. Detection of CTCs at different time points during the course of treatments as during, before, and after therapies enables prediction of therapeutic outcome [63]. Molecular characterization of CTCs would be helpful in predicting the response to treatment as demonstrated by Reinholz and colleagues [90]. They reported that the decrease in mammaglobin 1 mRNA expression in CTCs that were obtained from patients with metastatic breast cancer might assist in predicting the patient’s response to anti-cancer treatment.

## Gene expression signatures and applications in common cancers

Comprehensive understanding of carcinogenesis and hallmarks of cancer is increasingly important. Large-scale systematic sequencing endeavors have been implemented over the last decade [42]. The Cancer Genome Atlas and the International Cancer Genome Consortium have afforded the researchers and oncologists better focused insight into the complexity of cancer [91]. In particular, the medley of genomic, transcriptomic, and epigenomic aberrations are complicit in carcinogenesis [92]. In spite of the complexity of the cancer genome, solid tumors, such as breast, colon, prostate, and lung cancers, may result from a few mutations, and similarly, hematological malignancies may be caused by even fewer mutations [12], [93]. Cancer cells originating from the same tissue can possess distinct molecular characteristics and as a result, these differences can be applied for clinical prognosis and guide appropriate therapy [94]. Utilization of DNA microarray has led to comprehensive understanding of transcriptional activities in cancer cells. Moreover, molecular characterization of cancer cells will allow the implementation of new potential targets for cancer and development of new therapies and cancer biomarkers to monitor effectiveness of cancer therapy [95]. Lately, extensive genomic reports on many cancer types such as breast, colon, lung, ovarian, and renal cancers have been released. Their aims were to identify genomic alterations that may be potentially targetable or associated with drug resistance, thus enabling personalized cancer therapy [96]. The best example is the studies on the breast cancer genome, which identified potential targets for specific molecular alterations of breast cancer [97]. Females carrying mutations in genes related to DNA repair, such as *BRCA1* and *BRCA2*, had higher risk for breast cancer [68].

## Signatures of miRNA expression

miRNAs have attracted tremendous attention during the last decade especially in the implementation era of cancer gene targets and in the new cancer classification. Large-scale miRNA expression profiling “miRNAome” studies have potentially identified miRNA signatures that were applicable to multiple cancers. Specifically, these miRNAs have clear interactions with specific tumor suppressor genes or oncogenes in cancers of different tissue origins [98], [99]. For example, miR-17, miR-20, and miR-92 are usually associated with c-Myc during lymphoma development [100], whereas overexpression of miR-106a has been linked with downregulation of the tumor suppressor *RB1* in gastric, prostate, and lung cancers [99]. In addition, an inverse correlation has been observed between expression levels of miR-20a and *TGFBR2* [101].

Recently, miRNA has been extensively utilized as one of the promising predictive biomarkers through RNA expression analysis [102], such as studying expression of miR-342 as predictive biomarker for the response to tamoxifen in MCF-7 cell line. miR-342 is expressed only in cancer cells that are susceptible to tamoxifen therapy and blockage of its expression will lead to resistance to that chemotherapy [103]. Therefore, lower expression levels of miR-342 may point to resistance to tamoxifen therapy. Meanwhile, reversing miR-342 expression may be an effective response and therapeutic approach [103]. In the same context, miRNA may be considered as promising predictive and prognostic markers in bladder cancer [104], whereas deregulation of miRNA expression has been significantly related to dysplasia [104]. Among the studied miRNAs that are related to bladder cancer, expression of 15 miRNAs was strongly correlated with the responsiveness to cisplatin chemotherapy, whereas expression of five miRNAs was correlated with survival time. In addition, expression of three miRNAs, miR-886-3p, miR-923, and miR-944, has been correlated with response to chemotherapy and survival time [105].

## Breast cancer

Breast cancer can be grouped into four classes based on gene expression profiling [106], with each class defined by cohort of molecular biomarkers [107]. These include (1) luminal-A class that is estrogen receptor (ER)-positive and of low histological grade, (2) luminal-B class that is ER-positive with high histological grade, (3) basal-like class that is triple negative, *i.e.*, ER-negative, progesterone receptor (PR)-negative, and human epidermal growth factor receptor 2 (HER2, also known as erbB-2)-negative, and (4) HER2*-*like class that usually overexpresses *erbB-2* and is HER2*-*positive. These four classes could be differentiated by microarray studies. Luminal cancers show increased expression of genetic biomarkers reminiscent of normal breast myoepithelial cells, such as luminal cytokeratin, whereas basal-like cancers do not overexpress such genes or E-cadherin either [108]. Instead, basal-like cancers have been correlated with other markers, such as cytokeratin 5, c-Kit, hepatocyte growth factor, EGFR, and insulin growth factor [109]. In addition, most *BRCA1* mutation-associated breast cancers possess a molecular signature of basal-like cancers [110]. Lately, gene expression signatures by DNA microarray have been applied as a prognostic tool to determine the response to cancer therapy and to predict survival outcomes. A such example is MammaPrint (Agendia, Amsterdam, The Netherlands) assay that is composed of 70 genes and used to stratify patients into high or low risk groups [111]. Another example is the qRT-PCR based assay of Oncotype DX (Genomic Health, Redwood City, CA) [74] containing a 21-gene signature. Both assays may provide very effective prognostic information and would help physicians in selecting early stage hormone-responsive breast cancer patients that are likely to have disease recurrence [112]. Signatures for both assays include *ER*, *HER2*, PR-regulated transcripts, and proliferation-linked genes, which have been very effective in assessing the probability of breast cancer recurrence and classifying patients accordingly into high-, intermediate-, or low-risk groups for recurrence [111]. Moreover, Oncotype DX assay may be used for assessing response to tamoxifen or other modalities of adjuvant chemotherapy, such as fluorouracil [113], [114]. Invasiveness gene signature (IGS) is another gene signature for breast cancer involved in metastases. This signature is characterized in *CD44-*positive and *CD24-*negative patients [115]. Interestingly, IGS demonstrates a positive correlation for prediction of survival and free of metastasis interval not only for breast cancer patients but also for lung and prostate cancer patients, indicating that IGS may represent a generalized common feature for cancer cells of different origin [116].

## Colorectal cancer

There is not much known about the molecular alterations associated with CRC and no novel molecular biomarkers have been validated for clinical practice of diagnosis or prognosis. The multistep progression of CRC may involve changes of genes encoding KRAS, APC, P53, and mismatch repair (MMR), as well as proteins in transforming growth factor (TGF) pathway [87], [117]. Profiling studies of gene expression in CRC were conducted by comparing normal and tumor tissue samples at different stages of the disease. Using microarray, a 23-gene signature was identified to be a potential predictor for recurrence even in patients with negative lymph node and for relapse during disease-free survival time [118]. This signature may be utilized for upstaging the node-negative CRC patients to be directed to adjuvant anti-cancer therapy [119].

## Prostate cancer

Initiation and progression of prostate cancer is driven by genetic alterations and rearrangements that eventually lead to activation of oncogenes and inactivation of tumor suppressor genes [120]. These alterations most commonly include deletion of the tumor suppressor gene encoding the phosphatase and tensin homolog deleted on chromosome 10 (*PTEN*) and translocations of the genes encoding E-twenty-six specific (ETS)-related gene (*ERG*) and transmembrane protease, serine 2 (*TMPRSS2*), leading to the generation and increased transcription of the *TMPRSS2-ERG* fusion gene [121], [122]. Other common genetic changes include amplification of androgen receptor gene and *c-MYC*, as well as deletions of *CDH1*, *RB1*, *RAF*, *SMAD4*, and *NKX3-1* genes [123]. PCa gene 3 (*PCA3*) is a predictive biomarker that has been extensively studied recently. *PCA3* is a ncRNA that is highly specific for prostate tissue and overexpressed in PCa, indicative aggressive tumors. Gene fusions *TMPRSS2-ERG* and CTC counts, which have been shown to correlate with prognosis in castration-resistant PCa, could be used as predictive biomarkers, providing more personalized therapeutic alternatives to individual patients [124], [125].

## Lung cancer

A 5-gene signature profiling for lung cancer has been identified by combining microarray profiling and qRT-PCR and was implemented to predict overall survival and relapse-free survival time [126]. Similarly, a 12-gene signature was identified to be associated with lung cancer recurrence after surgical treatment [127]. This signature could stratify patients more effectively than the current staging system for lung cancer, such as stratifying grade 1B patients as being susceptible to adjuvant chemotherapy. In addition, this 12-gene signature can also predict chemo-sensitivity and/or chemo resistance to therapies involving carboplatin, cisplatin, erlotinib, paclitaxel, and gefitinib, which are commonly used in lung cancer treatment [128].

## Targeted therapy

Identical alterations or similar molecular signatures of certain cancers may lead to dissimilar manifestations in different individuals owing to tumor heterogeneity, thus leading to variations in the efficacy of diagnostic or prognostic markers, choice of potential cancer therapy, treatment outcome, and survival duration of two seemingly identical types of cancer [129]. Therefore, availability of diagnostic, prognostic, and predictive biomarkers of molecular alterations in each cancer would become a key trait for clinical practice in the future, to allow accurate and precise prediction of the patient’s response to therapy with proper stratification into particular groups, and defiant personalization of cancer treatment [23]. With personalized therapy, patients are treated according to their specificity of molecular profiles or signature characterizing such individual tumor tissue and preferentially by targeted therapy substances. HER2 blockade (trastuzumab) in HER2-positive breast cancer, tyrosine kinase inhibitor in CRC and chronic myelogenous leukemia, and inhibitors of EGFR in *EGFR*-mutated lung cancer are clinically established as examples of targeted therapy [130]. In fact, the response to currently used anti-cancer therapy may be variable for 10%−>90% of patients with different types of progressed cancer. On the other hand, the newly-targeted therapies may be highly effective only for few patients, demonstrating the clinical value of prospective identification of patients who perhaps will get a response to a specific targeted therapy [131]. A predictive biomarker that could differentiate between responders and non-responders to the targeted therapy could also identify cancer patients with high response rate and/or improved survival rate. This would help with informative treatment decision and outcome improvement for such cancer patients [132]. Targeted therapies for selected cancer, the targeted genes or receptors, and the predictive biomarkers used for assessment of their effectiveness are summarized in Table 3.

**Table 3:** **Targeted therapies for selected cancer and the predictive biomarkers used for efficacy assessment**

**Target**	**Drug**	**Cancer type and uses of targeted therapy**	**Predictive biomarker**	**Refs.**
HER2	Trastuzumab	First-line or adjuvant therapy for HER2-positive metastatic BC patients	Overexpression of *HER2*	[136], [200]
	Pertuzumab	First-line therapy for HER2-positive metastatic BC patients	Amplification of *HER2*	[201]

HER2; EGFR	Lapatinib	HER2-positive metastatic BC patients	Overexpression of *HER2*	[202], [203]
		ER, PR, and HER2 triple positive postmenopausal BC patients	HR-positive and HER2-positive	[204], [205]

EGFR	Cetuximab	EGFR-positive metastatic CRC patients	EGFR protein expression	[144], [206]
	Panitumumab	Metastatic CRC patients on chemotherapy and EGFR-positive CRC patients	Wild-type *KRAS*	[207], [208]
	Gefitinib	NSCLC patients with *EGFR* mutations	EGFR-activating mutations	[209], [210]
	Erlotinib	First-line therapy for metastatic NSCLC patients with *EGFR* exon 19 deletions or exon 21 mutations	*EGFR* deletion or mutation	[211], [212]

ALK	Ceritinib	*ALK*-positive NSCLC patients progressing during or after treatment with crizotinib	*ALK* rearrangement	[213], [214]

ALK	Crizotinib	*ALK*-positive locally-advanced or metastatic NSCLC patients	*EML4-ALK* translocation	[163]

*Note*: ALK, anaplastic lymphoma kinase; BC, breast cancer; CRC, colorectal tumor; EGFR, epidermal growth factor receptor; EML4, echinoderm microtubule associated protein like 4; ER, estrogen receptor; HER2, human epidermal growth factor receptor 2; HR, hormone receptor; KRAS, Kirsten rat sarcoma viral oncogene; NSCLC, non-small cell lung cancer; PR, progesterone receptor.

## Trastuzumab, pertuzumab, and lapatinib targeting HER2

Trastuzumab (herceptin) is an antibody therapy targeting HER2, which is amplified in about 10% of breast cancer by acting on its domain IV [133]. Pertuzumab (Perjeta) is an approved monoclonal antibody against ligand domain II of HER2. Lapatinib, a tyrosine kinase inhibitor (TKI), is another drug targeting both HER2 and EGFR [134]. Addition of trastuzumab to traditional adjuvant chemotherapy for HER2*-*positive breast cancer patients showed significant increase in overall survival and metastasis-free interval in two large American and European group studies [135], [136]. Lapatinib is also a TKI, which targets HER2 and possesses high affinity to the EGFR1 intracellular domains [137]. Lately, combined therapy of pertuzumab, trastuzumab, and docetaxel was approved for patients with invasive HER2-positive breast cancer before implementing such regimen [138]. There was strong evidence showing that combining two HER2 targeted therapies (trastuzumab, lapatinib or pertuzumab), no matter for metastatic or neoadjuvant settings, would improve patient’s outcome when compared to a single anti-HER2 therapy [139], [140], [141], [142]. This could be explained by the concomitant administration of synergistically-acting oncogene de-addiction agents, the higher benefit from blocking the same receptor by two different agents targeting two different sites, and more importantly, activation of the immune system in parallel to oncogene de-addiction that may involve immunogenic cell death [143].

## Imatinib, cetuximab, and panitumumab targeting EGFR

EGFR is a tyrosine kinase receptor and gets involved in the key signaling pathway for initiation and progression of CRC. Imatinib, cetuximab, and panitumumab, which are TKIs against EGFR, are used for the treatment of metastatic CRC. Earlier studies showed lower response rate in metastatic CRC patients with overexpressed *EGF R* when taking cetuximab as monotherapy than those taking the combined therapy of cetuximab and irinotecan [144]. In addition, imatinib was also used clinically to treat chronic granulocytic leukemia and gastrointestinal stromal tumor (GIST) patients carrying mutations in the gene encoding platelet derived growth factor receptor A (*PDGF-RA)* or oncogene *KIT* [145], [146]. Imatinib was speculated to achieve cancer control in GIST patients by targeting c-Kit. Additionally, imatinib may activate innate immune response [146] and lead to a sustained objective response and long-term stabilization of the patients [147]. Imatinib is used as the first-line treatment in patients with chronic myeloid leukemia, particularly those at early phase. It can inactivate the protein encoded by Abelson murine leukemia viral oncogene homolog 1 gene (*ABL1*) by competing with ATP at its binding site on ABL1, thus inhibiting kinase activity of ABL1 [145]. Imatinib has also been reported to inactivate other oncoproteins and their kinases such as PDGFR, cellular Rous sarcoma virus non-receptor tyrosine kinase (c-Src), Tec, and erythropoietin-producing hepatoma receptor tyrosine kinase (Eph) [148].

Cetuximab and panitumumab are also EGFR-targeted monoclonal antibodies used for treatment of metastatic CRC. However, prediction of the outcome of both drugs can’t be achieved by relying on positive expression of *EGFR* alone [149]. Clinical trials have reported that *KRAS* mutations could effectively predict resistance to both drugs [150]. Few patients carrying *KRAS-*mutated tumors were responsive to cetuximab and panitumumab treatment [151]. *KRAS* mutations, *EGFR* gene copy number variations, as well as *BRAF* and *PIK3CA* mutations, could also be useful for the prediction of patients with low response rate or secondary resistance to anti-EGFR therapy [152].

## Crizotinib targeting EML4-ALK

Numerous studies have demonstrated the inter-individual tumor heterogeneity of NSCLC [153], [154], [155], [156], [157]. NSCLC is classified into several subtypes according to the driving genomic alterations, such as mutations in the *BRAF*, *KRAS*, *HER2*, and *EGFR*, as well as translocations of the gene encoding anaplastic lymphoma kinase (*ALK)*, rearrangements of the gene encoding c-ros oncogene 1 receptor tyrosine kinase (*ROS*), and fusion of the gene encoding rearranged during transfection tyrosine kinase receptors (*RET*) [158]. So far, mutations of *EGFR* and translocations of the oncogenes encoding echinoderm microtubule-associated protein-like 4 (*EML4*)*-ALK* had been identified as the most common ones [159]. Potent targeted agents against any of the identified specific genomic alterations would definitely improve the outcome of NSCLC cancer treatment [160], [161]. Investigation of anti-*ALK* crizotinib (Xalkori), which targets *EML4-ALK* translocation, in patients with NSCLC showed promising results. 57% of patients responded to the drug treatment with 72% 6-month progression-free survival reported [162]. Crizotinib was approved in 2014 by the Food and Drug Administration (FDA), United States for clinical treatment of advanced or metastatic NSLC that is positive for *ALK* translocation [163].

## Resistance to targeted therapy

**Figure 3:**
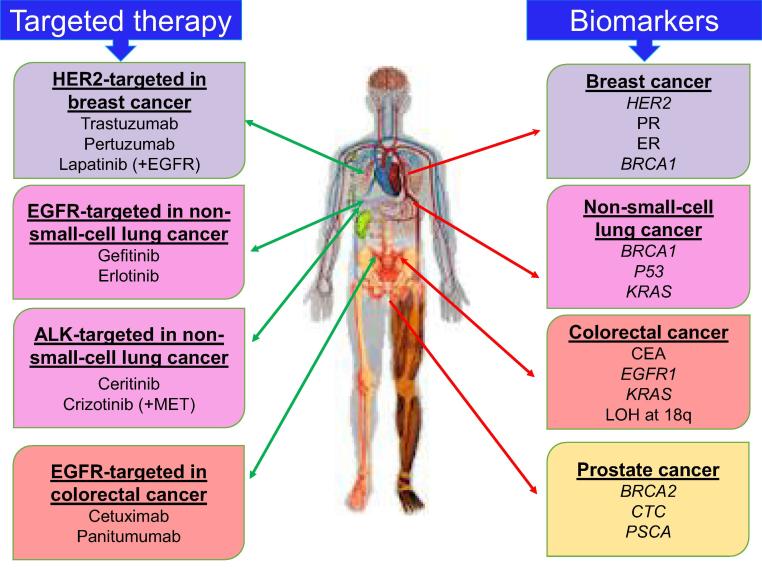
**Body map of currently available biomarkers and targeted therapies for different types of cancer** A body map for prognostic and predictive biomarkers used for assessment of response to targeted therapies in breast cancer, colorectal cancer, non-small cell lung cancer, and prostate cancer. HER2, human epidermal growth factor receptor 2; EGFR, epidermal growth factor receptor; ALK, anaplastic lymphoma kinase; PR, progesterone receptor; ER, estrogen receptor; BRCA1, breast cancer 1 gene; KRAS, Kirsten rat sarcoma viral oncogene; CEA, carcinoembryonic antigen; LOH, loss of heterozygosity; CTC, circulating tumor cells; PSCA, prostate stem cell antigen.

Therapies targeting oncoproteins, such as anti-HER2 (trastuzumab), c-Kit inhibitor (imatinib), and ALK inhibitor (crizotinib), have been successfully developed and extensively used recently [164]. However, oncogene de-addiction has been facing some challenges, such as the appearance of resistance to targeted therapies. It was proposed that drug resistance might result from secondary genomic events, genomic instability, as well as intratumor and intertumor heterogeneity [143], [165]. In addition, resistance may be due to alterations in the signaling pathways or in the feedback loops, just as in the case of cytotoxic chemotherapy [166]. Omics technology with recent longitudinal sequencing facilities for whole-exome sequencing may help oncologists to better understand the possible mechanisms underlying drug resistance, and consequently concomitantly administrate several targeted therapies that specifically match with the individualized profile for that tumor [167]. Additionally, omics technology facilitates the development of comprehensive panel of predictive biomarkers to better assess therapeutic response [168], [169]. Recently, using nanotechnology and oligoclonal nanobodies to target different epitopes on HER2 has been shown to be promising as one of the most efficient targeted system in nanomedicine [170]. A body map of currently available biomarkers and targeted therapies for different types of cancer is illustrated in Figure 3.

## Conclusion and perspectives

Recent advances in molecular diagnostics have offered essential tools allowing applications of personalized medicine in oncology. The fast development of sequencing and microarray methods may facilitate the emergence of new biomarkers and targeted therapies for individual cancer patients, paving the road toward the new era of personalized medicine. Comprehensive genomic studies on most of the common cancer types, such as breast, colon, lung, ovarian, and renal cancers, have been released recently. These studies aid in identifying most of the genomic alterations that may be targetable or associated with drug resistance, thus enabling individualized cancer therapy. Resistance to targeted therapies might be due to secondary genomic alterations or instability and intra-tumor heterogeneity. Therefore, overcoming resistance would involve the concomitant administration of targeted therapies and implementation of specifically employed panel of predictive biomarkers. Better comprehensive understanding and proper evaluation of the information offered synchronously by prognostic and predictive biomarkers would enable quicker and earlier diagnoses as well. Predictive markers are also beneficial in reducing the toxicity and resistance to treatments and in assessment of the suitability of patients to a targeted treatment, in another word, “individualized biomarker-driven cancer therapy” or “personalized medicine”.

Integrated omics studies provide better understanding and identification of the crucial molecules involved in cancer development and progression. In addition, Technological breakthrough in microarray, automated DNA and RNA-sequencing, mass spectrometry, comparative genomic hybridization, *etc.* enables the identification of promising prognostic and predictive cancer biomarkers for better assessment and follow up of cancer patients.

One of the major limitations of personalized medicine is the unique genomic profiling of each patient. In fact, the alterations in each gene occur with low incidence. Therefore, the medication development in cohorts driven by a genomic alteration is encountered by the low frequency of genomic alteration. To overcome this obstacle, scientists would need to develop genomic algorithms and implement highly specified and constructed software for personalized medicine, allowing prediction at the individual patient level.

## Competing interests

The authors have declared no competing interests.
